# Dr. O. L. Lloyd

**Published:** 1985-07

**Authors:** T. F. Hewer


### Obituary

### Dr. O. C. Lloyd

Oliver Cromwell Lloyd, who died unexpectedly at the end of May, came to the University of Bristol as a Lecturer in Pathology in 1948 and retired in 1977. Qualifying in 1936 from the London Hospital he chose a career in Pathology and held a Royal Society research appointment at the Radcliffe Infirmary in Oxford after working in the E.M.S. during the War. When he came to Bristol he had already a high reputation as a pathologist, specialising in morbid anatomy. His appointment to the Department of Pathology came at an important time in its post-war development, especially in relation to the teaching hospitals of the Bristol Royal Infirmary and the Bristol General Hospital. He quickly established himself as a most reliable and stimulating colleague and enjoyed the confidence of everyone in the hospitals so that the association between the University Department and the clinicians became very close and mutually successful. His work, for instance, with the surgical team that made great progress in the diagnosis and treatment of malignant melanoma was a perfect example of such cooperation.

As a teacher and a lecturer he was an inspiration to his students who enjoyed his eccentricities and came to realise the depth and quality of his knowledge that he imparted to them so vividly. Those of us who worked with him in the Department of Pathology profited by his opinion on almost any histological diagnostic problem. He was always ready to spend unlimited time to help any one and never sought recognition. He was a most remarkable and delightful man, excelling in many fields as well as that of his profession. When he came to Bristol he said his hobby was entomology and he was a Fellow of the Royal Entomological Society but he soon became a member of the student Spelaeological Society, using sophisticated methods of underwater exploration, often with the physical courage that made him a leader of the Mendip Cave Rescue Organisation. It was not until late in his retirement that he allowed his age and the need for a hip operation to restrict these often dangerous activities.

Music and acting were other activities of Oliver's. He took part in the student's Operatic Society and entertained them to musical evenings in his home. When he retired he joined the Department of Music as a part-time student specialising in composition.

People who knew him casually in the past probably remember him for some rather surprising sartorial peculiarities although nowadays they would cause little comment. Above all these characteristics was his capacity for friendship and his generosity in so many small ways. He was, for instance, keen on collecting edible fungi and knew where to find the more exciting and delicious ones. Some of us were often lucky enough to find on our doorstep a paper bag containing a present of some delicious ones: and knew they came from Oliver.

